# Population and contact tracer uptake of New Zealand’s QR-code-based digital contact tracing app for COVID-19

**DOI:** 10.1017/S0950268824000608

**Published:** 2024-04-17

**Authors:** Tim Chambers, Andrew Anglemyer, Andrew Chen, June Atkinson, Michael G. Baker

**Affiliations:** 1 Department of Public Health, University of Otago, Wellington, New Zealand; 2 Department of Preventive and Social Medicine, University of Otago, Dunedin, New Zealand; 3 Koi Tū: The Centre for Informed Futures, The University of Auckland, Auckland, New Zealand

**Keywords:** COVID-19, digital contact tracing, quick response code, Exposure Notification Framework, health equity

## Abstract

This study aimed to understand the population and contact tracer uptake of the quick response (QR)-code-based function of the New Zealand COVID Tracer App (NZCTA) used for digital contact tracing (DCT). We used a retrospective cohort of all COVID-19 cases between August 2020 and February 2022. Cases of Asian and other ethnicities were 2.6 times (adjusted relative risk (aRR) 2.58, 99 per cent confidence interval (95% CI) 2.18, 3.05) and 1.8 times (aRR 1.81, 95% CI 1.58, 2.06) more likely than Māori cases to generate a token during the Delta period, and this persisted during the Omicron period. Contact tracing organization also influenced location token generation with cases handled by National Case Investigation Service (NCIS) staff being 2.03 (95% CI 1.79, 2.30) times more likely to generate a token than cases managed by clinical staff at local Public Health Units (PHUs). Public uptake and participation in the location-based system independent of contact tracer uptake were estimated at 45%. The positive predictive value (PPV) of the QR code system was estimated to be close to nil for detecting close contacts but close to 100% for detecting casual contacts. Our paper shows that the QR-code-based function of the NZCTA likely made a negligible impact on the COVID-19 response in New Zealand (NZ) in relation to isolating potential close contacts of cases but likely was effective at identifying and notifying casual contacts.

## Key result

- Public uptake and participation in the quick response (QR) code system were high (45%).
- Ethnicity and contact tracing organizational level were the main predictors of whether location tokens were generated for cases.
- Public health official uptake of the QR code system was suboptimal (only 15% of cases with data utilized by Public Health Units (PHUs)).
- The positive predictive value (PPV) of the QR code system for identifying close contacts was close to nil but close to 100% for identifying casual contacts. Active consumer participation in the QR code system was closely correlated with perceived COVID-19 risk in the community.

## Introduction

Contact tracing is a key public health response measure to control infectious diseases, including severe acute respiratory syndrome coronavirus 2 (SARS-CoV-2) (which causes COVID-19) [1]. Digital contact tracing (DCT) technologies were implemented across the globe to assist with contact tracing [2]. These technologies have ranged from Bluetooth proximity apps, quick response (QR) code apps, and global positioning system (GPS) technology [3]. Previous research has primarily focused on the public uptake of these technologies [3], including New Zealand (NZ) [4–6]. To our knowledge, assessments of effectiveness have focused on the Bluetooth Exposure Notification Framework (ENF) [7–17], while there appear to be limited assessments of the effectiveness of the location-based QR code systems [18], with no assessments in the Western Pacific Region [3].

In NZ, the New Zealand COVID Tracer App (NZCTA) was released in May 2020 (see Supplementary Material for a full overview of the DCT developments in NZ). The NZCTA launched with the functionality to update an individual’s contact details and the ability to scan QR codes for location tracking (generally as a poster at the entrance of a location). In August 2020, the government announced it was mandatory for businesses to display a QR code poster; however, anyone could make and display a QR code poster for their location (e.g. private residence; public park). In September 2021, the scanning of QR codes for individuals was also made mandatory.

**Figure 1.**
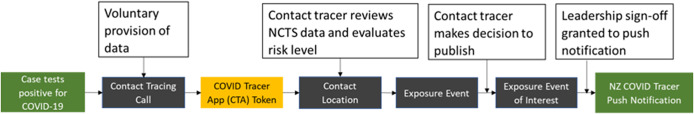
Overview of the data flow for the QR system of the New Zealand COVID Tracer App (NCTS=National Contract Tracing Solution).

Bluetooth functionality was introduced in December 2020 but is not covered in this paper. While there was substantial overlap in the time periods where both the location and Bluetooth systems were operational (December 2020 to February 2022), in practice there was substantial heterogeneity in the way the systems were utilized by the public health system and incorporated into government policy at different phases of the pandemic so we opted to assess these systems separately. The QR codes contained a unique location identifier and address information, which users would scan to create a log on their phone. The app then allowed contact tracers to send relevant locations and times (called exposure events) to all NZCTA users’ phones for comparison against the locally held diary, generating a notification if there was a match. Cases could also upload their diary if they were issued a location token to contact tracers during a case interview. After a location token was generated, there were a number of manual processes before notifications could be generated for potential contacts (outlined in Figure 1).

The majority of DCT evaluation studies have focused on the potential barriers and facilitators to the public adoption of these tools [3]. The research focus on public uptake is justified as modelling, and empirical studies have demonstrated that the effectiveness of these tools is highly dependent on coverage [9, 19]. However, the efficacy of DCT is also dependent on close contacts modifying their behaviour (e.g. isolating or getting tested upon notification). One evaluation in Switzerland of Bluetooth notifications estimated that from 1,374 close contacts, 939 followed up for testing, 722 called a Healthline, and 170 callers received a quarantine recommendation [8]. However, in Switzerland, the notification messages decreased in severity (e.g. from prioritized testing to prompt to call Healthline) and there was a parallel decrease in contact compliance over the pandemic (from 68% to 25%) [7, 8].

Limited research has focused on the public health sector adoption of these tools internationally, which is a strong determinant of the potential efficacy of these tools. One study of the SwissCovid Bluetooth app suggests compliance from public health officials was high (e.g. > 90%) [20]. However, in NZ, during the beginning of the pandemic in 2020, there was a documented reluctance from public health officials to adopt DCT tools [21] as well as other digital solutions developed for the contact tracing process [22, 23]. The main concerns around DCT tools were around false positives that may place an unnecessary burden on individuals and false negatives, which would lead to close contacts being missed.

The limited assessments of the sensitivity and positive predictive value (PPV) of DCT tools have primarily focused on Bluetooth-based proximity tracing and the identification of close contacts [9, 20, 24]. In evaluations including only app users (e.g. assuming 100% uptake in the population), the sensitivity in identifying close contacts in Australia was estimated at 15% based on 35 of 236 self-identified app users and close contacts of cases receiving a notification from the Bluetooth app [9]. Estimates in Switzerland ranged between 39% and 58% based on two separate cohort studies [20, 24]. The PPV relates to the proportion of identified close contacts that were actually close contacts. In Australia, 39% of identified contacts by the Bluetooth app were deemed clinically relevant, suggesting the COVIDSafe app had a PPV of 39%. The potentially low PPV of DCT tools has led to concerns around a ‘pingdemic’ characterized by a large number of consecutive notifications to potential contacts that may result in decreased compliance [12].

The stated purpose of the NZCTA QR code function was ‘for Consumers to record their movements so that if they become infected with COVID-19 they can quickly and accurately identify others who may be close contacts or casual contacts’ [25, p. 6]. In this context, using the United States Centers for Disease Control and Prevention (CDC) definition of sensitivity [26], sensitivity refers to how well a DCT tool can correctly identify a close or casual contact from all exposed contacts (as opposed to just app users) and therefore the likelihood that true contacts are not missed.

This paper seeks to (i) understand the public and contact tracer uptake of the QR code function of NZCTA throughout the COVID-19 pandemic and (ii) estimate the PPV of the QR code system for detecting close and casual contacts.

## Methods

### Study design

This study has two components. The first was a retrospective cohort study design using all diagnosed COVID-19 community cases (excluding international cases isolated at the border within managed quarantine facilities). Participants were any diagnosed COVID-19 case aged 15+ in NZ stored in the National Contact Tracing Solution (NCTS), a centralized information technology (IT) platform to support the end-to-end contact tracing process. Our observation period was from August 2020 (the date data were available in NCTS) to 16 February 2022 (which marked the effective end date for the elimination/suppression phase of the NZ COVID-19 response and transition to a mitigation response with the majority of contact tracing switching to a self-service model after that date).

The second component was a descriptive analysis of the NZCTA uptake data.

### Data sources

Contact tracing data were sourced from the Ministry of Health. The data provided include three main data sets. First, the NCTS data include anonymized information about all community COVID-19 cases in NZ at the individual level including information on case ethnicity, age, sex, contact tracing organization (either a Public Health Unit (PHU) or the National Case Investigation Service (NCIS)), and whether a contact tracer generated a location token for the case. A token is a digital key that provides (or unlocks) the opportunity for a case to upload their data, and without this token, a case is unable to upload their data validation tool (DVT) data even if they are willing. Second, NZCTA location data include all QR code-derived locations of interest (which were uploaded by a case), exposure events (a location of interest that was prioritized by contact tracers), and pushed location (exposure events that were sent as push notifications to potential contacts). The NCTS and NZCTA data are linked at an individual level, although the recipients of notifications are not identifiable. Third, the NZ COVID-19 outbreak report contains data on the usage of NZ COVID Tracer notification functions on a daily basis (a full data dictionary is provided in Supplementary Material).

### Cohort study outcomes

Our primary outcome to measure contact tracer uptake was location token generation as a binary outcome. Token generation indicates that cases were given the opportunity to provide DCT data by entering the token/code into NZCTA, but it does not guarantee that the data were provided or that contacts were found or notified. Other outcomes include the number of push notifications that were sent out to contacts; the time delay between a case being entered into NCTS and a push notification being sent; and the associated risk messaging tied to those notifications.

### Analytical phases of the pandemic

NZCTA data were available from 12 August 2020 to 16 February 2022 before the self-service period began, representing the total extent of our observation period. We conduct our analyses separately for three time periods: (i) pre-Delta wave: 12 August 2020 to 16 August 2021; (ii) Delta wave: 17 August 2021 to 6 January 2022; and (iii) Omicron wave: 7 January 2022 to 15 February 2022. The self-service phase started officially on 16 February 2022 and was excluded from the current analysis. The self-service phase was defined by the transfer of the primary responsibility for contact tracing from contact tracers to individual cases through an online survey that was sent to cases via text message.

### Cohort study covariates that were investigated

Data from NCTS included case demographics of age, sex, and ethnicity. We classified age into four categories: 15–24, 25–44, 45–59, and 60+. Ethnicity was prioritized meaning a case was allocated to a single ethnic group in order of priority: Māori, Pacific, Asian, and European/Other. Data also indicate the contact tracing organization responsible for the case. Initially, contact tracing was managed by the 12 PHUs. Increasing case volumes associated with the Delta outbreak led to the NCIS being established in November 2021 [27]. NCIS contracted a call centre that specializes in health research to conduct contact tracing in November 2021. NCIS call centre staff were provided with training and a script to support contact tracing, including standard operating procedures (SOPs) around NZCTA uploads. A key distinction is that NCIS call centre staff were not necessarily clinically trained and therefore did not exercise clinical judgement in contact tracing decisions and were required to follow the scripts and SOPs provided. In our analysis, we collapse all PHUs into a single category, but the majority of the cases were handled by the Auckland Regional Public Health Service (ARPHS). As such, contact tracing organization represented either a PHU or NCIS.

### Public uptake

To estimate public uptake of the QR code system, we used the proportion of cases having a token generated by contact tracers from NCIS. As mentioned above, NCIS did not apply any clinical judgement on whether or not to ask a case for their NZCTA data so it was assumed that close to every single case handled by NCIS was provided an opportunity to upload their data. In contrast, PHU staff had discretion when they asked for NZCTA data and the data used in this research showed they applied this discretion frequently, and as a result, token generation by PHU does not reflect public uptake of NZCTA.

Publicly available NZCTA usage statistics also do not provide an accurate proxy for public uptake as the numerator only included the number of ‘active devices’ on any given day (e.g. devices making at least one scan that day). Consequently, on any given day, there could be a substantial proportion of the population that had NZCTA installed (uptake) but did not scan either because they did not go to a location of interest or they forgot to scan, which is not reflected in the ‘active devices’ statistic. As a result, the proportion of cases uploading tokens often far exceeded the proportion of the population with active devices on a given day demonstrating public statistics were not an accurate proxy of public uptake.

### Positive predictive value

To estimate the PPV of the QR code system of NZCTA for close contacts, we compared the notifications sent per location pushed against the median number of close contacts in the only documented peer-reviewed literature on close contacts identified by the contact tracing system in NZ (median = four close contacts), which occurred during an outbreak in Auckland in 2020 [28]. For casual contacts, by definition, every person who received a notification is likely a casual contact ‘any person with exposure to the case who does not meet the criteria for a close contact’ [29].

### Statistical analysis

Data cleaning, manipulation, and the production of descriptive statistics were conducted in SAS (version 9.4) and R (R-Project, www.r-project.org). We used a modified Poisson regression to estimate the effects of each predictor on location token generation [30]. A purposeful selection of covariates was used to develop initial multiple regression models [31, 32]. Full models were populated with all significant predictors (*p* < 0.1) from the univariate models, and backward elimination using Akaike’s information criterion (AIC) was used to help select the final model [33]. We calculated adjusted relative risks (a*RRs*) and their respective 95 per cent confidence intervals (CIs) for each included predictor in the multivariable models. All analyses were performed in R.

### Ethics approval

This study received a ‘Minimal Risk Health Research – Audit and Audit related studies’ research determination by the University of Otago Ethics Committee and was approved under application HD22/080.

## Results

### Cohort study participants

Table 1 shows the characteristics of the COVID-19 cases included in our retrospective cohort. Overall, Māori and Pacific people were over-represented in case numbers during the pre-Delta and Delta phases as well as overall compared to their population distribution. Asian cases were over-represented during the Omicron phase, while cases of other ethnicities were underrepresented across each period and overall. PHUs handled the most cases overall (61.8%), including during the pre-Delta (100%) and Delta (89.7%) phases. In the Omicron phase, NCIS handled the majority of cases (75.8%).

**Table 1.** Retrospective cohort of COVID-19 cases in New Zealand from August 2020 to February 2022

	Total	Pre-Delta	Delta	Omicron
Characteristics	N(%)	N(%)	N(%)	N(%)
All	13,958	176 (1.3)	7,820 (56.0)	5,962(42.7)
Sex				
Female	7,075 (50.7)	97 (55.1)	3,913 (50.0)	3,065 (51.4)
Male	6,875 (49.3)	79 (44.9)	3,903 (49.9)	2,893 (48.5)
Missing	8 (0.1)	0 (0.0)	4 (0.1)	4 (0.1)
Age				
15–24	3,764 (27.0)	40 (22.7)	2036 (26.0)	1,688 (28.3)
25–44	6,425 (46.0)	66 (37.5)	3,674 (47.0)	2,685 (45.0)
45–59	2,641 (18.9)	54 (30.7)	1,473 (18.8)	1,114 (18.7)
60+	1,128 (8.1)	16 (9.1)	637 (8.1)	475 (8.0)
Ethnicity				
Māori	4,011 (28.7)	30 (17.0)	3,280 (41.9)	701 (11.8)
Pacific	4,762 (34.1)	85 (48.3)	2,326 (29.7)	2,351 (39.4)
Asian	2034 (14.6)	29 (16.5)	475 (6.1)	1,530 (25.7)
Other	2,997 (21.5)	32 (18.2)	1,688 (21.6)	1,277 (21.4)
Missing	154 (1.1)	0 (0.0)	51 (0.7)	103 (1.7)
Contact tracing organization				
Public Health Units	8,630 (61.8)	176 (100.0)	7,011 (89.7)	1,443 (24.2)
National Case Investigation Service	5,328 (38.2)	0(0.0)	809 (10.3)	4,519 (75.8)

### Public uptake and active participation

**Figure 2.**
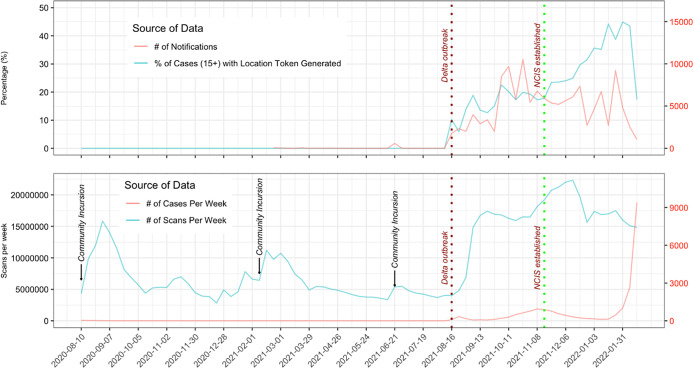
Percentage of cases with location token generated and number of location notifications (top) and number of COVID-19 cases per week and New Zealand COVID Tracer App scans per week (bottom). *Community incursions: On *11 August 2020,* four of the new cases are in the community. It was 102 days since the last case that was acquired locally from an unknown source; on *14 February 2021,* Auckland was put into Alert Level 3 lockdown at 11.59 pm after three cases were detected in the community in South Auckland; on *22 June 2021,* quarantine-free travel to New South Wales was suspended after 10 new community cases were reported in NSW; and on *23 June 2021,* the Wellington Region was put into Alert Level 2 at 6 pm following the visit of an Australian man who tested positive after returning to Sydney.

The top panel of Figure 2 shows the percentage of cases with location tokens generated and the number of location notifications sent from NZCTA per week. The proportion of cases uploading a location token substantially increased after the Delta outbreak and again after the establishment of NCIS. The number of location notifications sent out per week reached a peak midway through the Delta wave until levelling out and dropping off during the Omicron wave. The bottom panel of Figure 2 shows the number of COVID-19 cases per week and the number of NZCTA scans per week. Figure 2 highlights that the number of scans was highly dependent on the public perception of the risk of COVID-19 in the community. Prior to the Delta outbreak, there were three significant community incursions of COVID-19. Scans increased immediately after public notification of these events, including after the Delta outbreak. At peak usage of NZCTA during December 2021, there were almost 4 M QR code scans a day coming from approximately 1.45 M devices.

### Token generation and public uptake

Table 2 shows the regression results investigating differences in token generation during the Delta and Omicron periods. Contact tracing organization allocation was an influential factor for location token generation, with cases allocated to NCIS twice (aRR 2.03, 95% CI 1.79, 2.30) as likely during Delta and 1.5 times (aRR 1.51, 95% CI 1.34, 1.71) more likely during Omicron to generate a token than cases handled by PHUs. Overall, 45% of cases allocated to NCIS received a token compared to only 15% of cases allocated to PHUs. We use the token generation for NCIS as our estimate of public uptake of the location-based function of the NZCTA.

**Table 2.** Regression of location token generation in the Delta and Omicron periods by socio-demographic characteristics and contact tracing organization allocation

	Delta				Omicron			
	Total cases	Token generated	Crude estimate	Adjusted estimate^a^	Total cases	Token generated	Crude estimate	Adjusted estimate^a^
Characteristic	N	n (%)	RR (95% CI)	aRR (95% CI)	N	n (%)	RR (95% CI)	aRR (95% CI)
All	7,820	1,556 (19.9)	—	—	5,962	2,455 (41.2)	—	—
Ethnicity								
Māori	3,280	474 (14.4)	Ref	Ref	701	160 (22.8)	Ref	Ref
Pacific	2,326	343 (14.7)	1.02 (0.89–1.17)	1.04 (0.90–1.19**)**	2,351	787 (33.5)	**1.47 (1.24–1.74)**	1.13 (0.93–1.36)
Asian	475	222 (46.7)	**3.23 (2.75–3.79)**	**2.58 (2.18–3.05)**	1,530	812 (53.1)	**2.33 (1.97–2.76)**	**1.75 (1.45–2.11)**
Other	1,688	497 (29.4)	**2.04 (1.80–2.31)**	**1.81 (1.58–2.06)**	1,277	642 (50.3)	**2.20 (1.86–2.63)**	**1.79 (1.49–2.16)**
Unknown	51	21 (—)	—	—	103	54 (—)	—	—
Age								
15–24	2036	305 (15.0)	Ref	Ref	1,688	611 (36.2)	Ref	Ref
25–44	3,674	797 (21.7)	**1.45 (1.27–1.65)**	**1.28 (1.12–1.46)**	2,685	1,276 (47.5)	**1.31 (1.19–1.45)**	**1.25 (1.13–1.38)**
45–59	1,473	332 (22.5)	**1.50 (1.29–1.76)**	**1.31 (1.12–1.53)**	1,114	461 (41.4)	**1.14 (1.01–1.29)**	1.10 (0.98–1.25)
60+	637	122 (19.2)	**1.28 (1.03–1.57)**	1.00 (0.80–1.23)	475	107 (22.5)	**0.62 (0.50–0.76)**	**0.60 (0.48–0.73)**
Sex								
Male	3,903	679 (17.4)	Ref	Ref	2,893	1,179 (40.8)	Ref	Ref
Female	3,913	877 (22.4)	**1.29 (1.17–1.42)**	**1.37 (1.24–1.52)**	3,065	1,276 (41.6)	1.02 (0.94–1.11)	1.07 (0.99–1.16)
Unknown	4	0 (0.0)	—	—	4	0 (—)	—	—
Contact tracing organization								
Public Health Unit	7,011	1,187 (16.9)	Ref	Ref	1,443	397 (27.5)	Ref	Ref
National Case Investigation Service	809	369 (45.6)	**2.69 (2.39–3.02)**	**2.03 (1.79–2.30)**	4,519	2058 (45.5)	**1.66 (1.49–1.85)**	**1.51 (1.34–1.71)**

*Note:* Bold values represent statistically significant *p* < 0.05 modified Poisson regression.

a Mutually adjusted for ethnicity, age, sex, and Public Health Unit.

Cases of Asian and other ethnicities were 2.6 times (aRR 2.58, 95% CI 2.18, 3.05) and 1.8 times (aRR 1.81, 95% CI 1.58, 2.06) more likely than Māori cases to generate a token during the Delta period, which continued during the Omicron period. In total, 71.2% and 55.5% of Asian cases handled by NCIS generated a location token during the Delta and Omicron waves, respectively (see Supplementary Table 1). Cases aged 25–44 were 1.3 times (aRR 1.28, 95% CI 1.12, 1.46) more likely than cases aged 15–24 to generate a token during Delta and Omicron periods. Cases aged 45–59 were 1.3 times (aRR 1.31, 95% CI 1.12, 1.53) more likely to generate a token than cases aged 15–24 during Delta, but no significant difference was observed during Omicron. There was no statistically significant difference between cases aged 60+ and cases aged 15–24 during Delta, but during Omicron, cases aged 60+ were less likely to generate a token (aRR 0.60, 95% CI 0.48, 0.73) than cases aged 15–24. Female cases were 1.4 times (aRR 1.37, 95% CI 1.24, 1.52) more likely to generate a location token than male cases during Delta, but there was no difference observed during Omicron. Supplementary Figure 1 shows the proportion of tokens generated by socio-demographic characteristics and contact tracer allocation across the pandemic.

### Notification prioritization, positive predictive value, and processing time

Table 3 provides an overview of the location prioritization and PPV of the QR code system of the NZCTA. In total, 2,287 cases (16.6% of all cases) had a token generated and location data uploaded to NCTS, meaning that 12.1% of all cases had a token generated but produced no locations of interest. Reasons for no location data being uploaded include a case deciding not to upload data once being provided a token or a case not having a location recorded in the NZCTA. Only 298 cases had locations that were eventually pushed as an exposure notification (2.2% of all cases or 13.0% of all cases with tokens).

**Table 3.** Assessment of location prioritization of the QR code system of the New Zealand COVID Tracer App

	Overall^a^		Delta		Omicron	
Level of analysis	n	%	N	%	n	%
Case level						
Cases	13,782	100%	7,820	100%	5,962	100%
Cases with tokens	2,287	16.6%	1,009	12.9%	1,278	21.4%
Case with a location pushed	298	2.2%	208	2.7%	90	1.5%
Location level						
Contact locations	13,401	100.0%	6,328	100%	7,073	100%
Exposure events	2,714	20.3%	1,695	26.8%	1,019	14.4%
Locations pushed	844	6.3%	643	10.2%	201	2.8%
Notification level						
Notifications sent	137,738	–	106,968	–	30,770	–
Average notifications per pushed location	164	–	166	–	153	–

a Excluding the 176 cases from the pre-Delta phase.

Overall, 13,401 contact locations were uploaded to the NCTS, with 2,714 contact locations being upgraded to an exposure event by a contact tracer (20.3%). Upgrading a contact location to an exposure event means that the location could be further prioritized to be sent as a push notification to potential contacts (via NZCTA). Only 844 (31%) exposure events were prioritized to a push notification. The main locations that were prioritized to a push notification were classified as other (n = 325), retail store (n = 134), supermarket (n = 169), and contact location (n = 101) (for a breakdown of the exposure events that were prioritized to push notification by setting, see Supplementary Table 2). As a result, only 6.3% of all contact locations recorded by cases through the NZCTA were prioritized to a push notification sent to potential contacts.

In total, 137,738 notifications were sent to potential contacts, with an average of 164 notifications sent per location pushed. Given that the median number of close contacts detected per case during the pandemic was between four and eight across demographic groups [28], the PPV of the QR-based system for detecting close contacts was close to nil. In contrast, given the broad definition of a casual contact adopted by the Ministry of Health in NZ, it is likely every person receiving a notification was a casual contact meaning the PPV for casual contacts would be close to 100%.

To investigate the time required to undertake the manual processes outlined in Figure 1 after token generation, we calculated the time between the creation of a contact location in NCTS (e.g. a case uploads its data) and a push notification being sent (e.g. to alert potential contacts). Across the 836 cases with valid timestamps where the contact location creation precedes the push notification, the median time to push a location was 23.8 h (interquartile range (IQR) 16.8–42.7). In total, 687 (82%) push notifications were sent within 48 h.

### Risk messaging

The risk messaging in notifications changed over time. There were a total of 844 locations that were prioritized for push notifications. Of these, the risk messaging for 690 (81.8%) included a variation of the call to action to monitor your health or symptoms, and if unwell, stay home and call Healthline, for example ‘If you have been symptomatic or feel unwell, stay at home and call Healthline on 0800 3,585,453’. A further 18.2% (n = 154) of notifications made more direct instructions to self-isolate and/or get a test or call Healthline for further advice, for example ‘Self-isolate, test immediately and on day 5 after you were exposed at this location of interest. Further isolation and testing requirements will be provided by Public Health’.

## Discussion

Our evaluation of the QR code function of the NZCTA has shown that public uptake of the app was remarkably high, while contact tracer uptake among clinically trained staff at PHUs was lower than that among NCIS staff. The differential utilization of the QR code location data by PHU and NCIS staff in combination with a prioritized case allocation system led to higher Māori and Pacific caseloads for PHUs and thus inequities in the utilization of QR code data. Only a small proportion of cases providing their QR code data had it prioritized to a push notification to alert potential contacts. The manual processes for this prioritization took a median time of 23.8 h (IQR 16.8–42.7). On average, each pushed location sent out 164 notifications to potential contacts with the main call to action being to monitor symptoms.

The public uptake of the NZCTA was very high (45% of cases handled by NCIS uploaded tokens) compared to other countries with uptake rates between 20 and 30% [7, 10, 12, 34]. However, one limitation of the QR-code-based system is that it requires active participation or compliant adoption from individuals. In one NZ survey, only 40% of NZCTA users reported using it frequently, while 32% used it sometimes and 28% had installed but not used it [35]. Active participation was closely associated with perceived COVID-19 risk in the community. As a result, in the weeks prior to an outbreak, which data are most valuable to contact tracers during the outbreak, active participation was often at its lowest [6]. If active participation is reliant on active cases in the community, the NZCTA location histories of cases at the beginning of any outbreak are likely to be the most incomplete, leading to undetected contacts and further transmission. In contrast, other tools such as Apple–Google’s Bluetooth ENF work passively in the background once they are installed, which substantially mitigates the issue of compliant adoption.

One of the largest determinants of cases uploading location data or full participation in the system was the contact tracing organization allocation. The initial case triage system for cases resulted in the majority of Māori and Pacific cases being allocated to PHUs, while all other cases were assigned to NCIS, leading to an over-representation of Māori and Pacific cases handled by PHUs. An earlier 2020 study in NZ highlighted that PHU staff held substantial concerns around the usefulness of the DCT data [21]. It is possible that this perception persisted and led to a reluctance of PHU staff to consistently utilize the data. In contrast, NCIS staff were not clinically trained and were provided a script to adhere to, which included SOPs around NZCTA.

The differential utilization of QR code data by contact tracing organizations exacerbated existing inequities in access to the system by socio-demographic characteristics. For example, the adjusted rate ratio for token generation by ethnicity showed that Māori and Pacific cases were still less likely than other ethnicities to upload their data, suggesting a baseline level of inequity. This finding is consistent with the evidence in NZ, which suggests people living in higher deprivation or rural communities, people with disabilities, and Māori and Pacific peoples suffer disproportionately from digital exclusion [36]. Measures to address potential inequities driven by the NZCTA include prioritization of Māori and Pacific cases to clinically trained staff to ensure improved health service provision and support, but they also create inequities in access to the NZCTA system.

On average, each pushed location resulted in 164 notifications being sent to potential contacts. The only published research on the contact tracing system in NZ suggests the median number of close contacts identified per case was 4 [28]. The difference between the average number of notifications sent via NZCTA and the close contacts identified by contact tracers suggests that a large majority of those notified contacts were not clinically significant close contacts – resulting in a PPV for close contacts close to nil. In contrast, the PPV for casual contacts was likely closer to 100% given the broad definition of casual contacts adopted in NZ. The changing risk messaging throughout the pandemic (from self-isolation, to testing, to monitoring symptoms) likely reflects a shift in the original aim outlined in the privacy impact assessment of the QR-code-based system from identifying close and casual contacts to primarily identifying casual contacts (as close contacts were required to isolate). Thus, the utility of the QR code system is related to the value of identifying and isolating casual contacts, which is dependent on the characteristics of the pandemic (e.g. high transmissibility, high clinical severity, low controllability) [37] and associated policy responses (e.g. elimination strategy compared to a mitigation strategy) [38]. In the NZ context, the value of the QR code based system was likely higher when the country was pursuing an elimination strategy (which included closed borders, snap lockdowns, and an intensive testing and trace programme) but of marginal value after the shift to a mitigation strategy – reflected in the decision to cease QR code notifications in 2022.

Evaluations of DCT tools often implicitly assume that the manual system has a PPV of 100%. Our preliminary analyses of the manual contact tracing system in NZ showed there was wide variability in the definition and coding of close contacts across cases during this current observation period. For example, 5,551 cases were reported as having zero close contacts, while 258 cases had more than 50 close contacts each, with a maximum of 3,851 for one case. Unfortunately, the data provided to us are too unreliable to make any meaningful interpretation of what the manual contact tracing system achieved in terms of contacts traced per case, but it is a useful reminder that evaluations of DCT tools should be done in the context of the capacity and performance of the manual contact tracing system.

Other factors affecting the potential impact of the QR-code-based location system of NZCTA were the time required for the manual processes and the risk messaging provided to potential contacts. Firstly, the median time from uploading location data to a push notification was 23 h. The performance metric for the contact tracing system focuses on close contacts being traced within 48 h (P004) [39]. In combination with the delays between case identification and uploading contact locations (median 5 h, IQR 2.5–16) and push notifications being sent and received by a contact (unknown), it is likely most push notifications reached contacts beyond the 48-h target. Secondly, the majority of modelling evidence on the potential effectiveness of DCT tools was based on contact either isolating or taking a test [19]. However, the majority of notifications sent to potential contacts contained instructions to monitor symptoms with no call to isolate or get tested. It is unlikely these messages substantially influenced individuals to change their behaviour above and beyond what was accomplished via the extensive public health messaging that occurred external to the NZCTA.

### Strengths and limitations of this study

This study had a number of strengths including using national data at the individual level to evaluate the use of the QR-based aspect of the NZCTA. In particular, our study provides a unique insight into the utilization of a DCT tool by public health officials and contact tracers, which is often implicitly assumed to be near 100%. It also gave some preliminary estimates of PPV and the timeliness of this surveillance system.

Our study also had a number of limitations. First, it was hard to quantify accurately the final public uptake and compliant adoption of NZCTA. The usage statistics published by the Ministry of Health only account for unique devices used on any given day so people who simply did not scan or forgot to scan on a day were not counted. Furthermore, we cannot estimate what proportion of locations were not scanned among participating cases (another factor related to compliant adoption). Reliance on case data (NCIS in this case) to estimate public uptake has limitations as cases may have a different propensity to download and use NZCTA compared to individuals who were never infected or never tested. Second, we cannot determine why contact tracers did not consistently ask for or provide cases the opportunity to upload their NZCTA data. We have relied on data collected from 2020 to inform our discussion, but it is possible these attitudes and perceptions may have changed, which could be drawn out in an updated qualitative analysis. Third, we could not calculate the sensitivity of the QR code function of NZCTA. The main factors impacting the realized sensitivity of the tool were (i) public uptake (those without the app could not be detected); (ii) compliant adoption (those not scanning in at locations could not be detected); (iii) proportion of contact events occurring at non-participating locations (e.g. at home or other private residences); and (iv) problems with QR code implementation (e.g. many locations used unique QR codes for different areas of a single location meaning that people could scan in at different doors and thus not be detected).

One final limitation of our study is that we are implicitly focusing on one proposed benefit of DCT tools, which is to identify and notify close contacts of cases to increase timely isolation or testing. However, there are other potential benefits of the NZCTA that have been shared with us in qualitative interviews with members of the community and health officials. For example, health officials have commented that the NZCTA locations being fed directly into NCTS, rather than manually entered during case investigations, saved contact tracer time and created system efficiencies. This may be one reason location generation (18.4%) was so much higher than Bluetooth token generation (1.3%) within cases handled by PHU. It is highly unlikely that the difference between location and Bluetooth generation within PHUs is due to the differences in socio-demographic characteristics of cases handled by PHUs and their access or aversion to Bluetooth functionality. Health officials also said the public display of QR code posters increased public consciousness of COVID-19 risk and understanding of contact tracing, which helped contact tracers during case investigations. However, these potential benefits were outside the scope of this current analysis and are not the primary purpose of DCT tools.

## Conclusion

Our paper shows that the QR-code-based function of the NZCTA likely made a negligible impact on the COVID-19 response in relation to isolating or testing potential contacts of cases. Key factors influencing this conclusion include public access to full participation in the system being substantially impacted by contact tracer utilization of the NZCTA data; the delays built into the manual system from case creation to push notification decisions; and the risk messaging that was provided to contacts (e.g. to monitor symptoms). In the case of the QR code system, the value of this technology was primarily around identifying and notifying casual contacts, which likely had a greater importance when NZ was pursuing an elimination strategy compared to later in the pandemic.

A wider discussion is needed about the future role of QR code contact tracing. There are specific scenarios where this technology might be considered, notably to support the control of future epidemics and pandemics of (presumably respiratory) infectious diseases transmitted between people in specific settings. Such a discussion should commence now as part of pandemic preparedness. It would need to consider the relative benefits and costs of this technology versus Bluetooth and other DCT tools. Such systems should have quality assurance and evaluation features built into them so they can measure critical performance attributes such as sensitivity, PPV, timeliness, and equity. Whatever approaches are considered will require consultation with the wider health sector and community to ensure maximal participation in the system.

## Supporting information

Chambers et al. supplementary material Chambers et al. supplementary material

## Data Availability

The data that support the findings of this study are available from the New Zealand Ministry of Health. Restrictions apply to the availability of these data, which were used under license for this study. Data are available from the authors with the permission of the New Zealand Ministry of Health.

## Supplementary material

For supplementary material accompanying this paper visit http://doi.org/10.1017/S0950268824000608. click here to view supplementary material
