# Supplementary material for: Population and contact tracer uptake of New Zealand’s QR-code-based digital contact tracing app for COVID-19
Source: Epidemiol Infect. 2024 Apr 17;152:e66. doi: 10.1017/S0950268824000608 (PMC11062780; doi:10.1017/S0950268824000608)

# Supplementary material

## Appendix 1 Digital contact tracing developments in New Zealand

### Initial App Release and QR Codes

On May 20 2020, the Ministry of Health released the NZ COVID Tracer app (NZCTA). The app was available for smartphones (Android 7.0 or iOS 12 at launch) through the official app stores. The app launched with the functionality to update an individual’s contact details (as the National Health Index database is not always up-to-date) and the ability to scan NZCTA QR codes for location tracking.

These QR codes contained location information such as the address and a unique location identifier, which could be stored in a digital diary with the time in the app. The diary would automatically delete entries after 60 days, and they stayed on the device until they were voluntarily uploaded upon request of a contact tracer. This meant that data collection was decentralised, and only data for relevant individuals would be accessible to public health officials, helping to protect privacy. Initially, the location information would be manually processed by contact tracers and then published on a website – a later version allowed for contact tracers to send relevant locations and times (“exposure events”) to all apps for comparison against the locally-held diary, generating a notification if there was a match. The initial version of the app did not have a proximity detection function.

NZCTA was released a few weeks after New Zealand came out of strict ‘lockdown’ (stay at home order), at which point there were over 30 privately developed “check-in” or “sign-in” apps in use.^1^ These apps varied in design and approach, from scanning QR codes to digital forms, with both centralised and decentralised data storage, and sometimes collecting different types of personal information. Importantly, there were many other QR code formats that were not compatible with NZCTA, and businesses were free to choose which systems they supported. The initial fragmentation was problematic, as it led to significant confusion and usability challenges for end users.

### Second Wave (12 Aug 2020 to 6 Oct 2020)

In mid-August 2020, after the discovery of four cases of community transmission in Auckland, New Zealand was placed into a second lockdown. The government also announced that all businesses would be required to display a NZCTA QR code in a prominent place within the next week. Scanning the QR code was still optional for the consumer, but it would be mandatory to make a NZCTA QR code available. This new requirement caused the majority of private developers to turn off their systems and ask their business customers to switch to the government QR code. The efficacy of this policy change was evidenced in the number of QR codes being scanned per day – from approximately 20,000 scans per day before the lockdown, to over 2 million per day in the weeks after movement restrictions were loosened.

### Bluetooth Tracing (December 2020 to present)

In December 2020, Bluetooth was added to NZCTA. This was a proximity detection system based on the Apple/Google Exposure Notification Framework (ENF). This system used Bluetooth to exchange privacy-preserving identification keys wirelessly between enabled smartphones. If a user tested positive for COVID-19, contact tracers could ask them to upload their keys, which would then be broadcast to all devices, which could check locally if they had seen those keys recently, and (after applying threshold tests) notify the user that they may have been exposed.

Importantly, this approach only required users to enable it, and then it would run passively in the background (in contrast to the QR code scanning approach which required users to actively participate in the process). The ENF system was used in many other jurisdictions, and the NZCTA system was built from open-source code from Ireland’s implementation (COVID Green).^2^ Notably, the proximity detection system used in NZ was different to the one used in nearby jurisdictions Singapore and Australia, which adopted a centralised approach.

NZCTA therefore essentially had two systems running in parallel: QR code scanning for location tracking, and Bluetooth for proximity detection. In a contact tracing context, it is important to note that the QR code system provided information to human case investigators and contact tracers, who could analyse the information and apply their own risk assessments before acting (i.e. “augmenting” manual contact tracing), whereas the Bluetooth system ran more autonomously and was intentionally designed to prevent contact tracers from accessing identifying information.

For both location information and Bluetooth, the data lived on the device and was not automatically transferred to public health officials. If a person tested positive, then they could be given short codes to enter into NZCTA, one for location and one for Bluetooth, which would then release that data. For location information, it would go to the National Contact Tracing Service (NCTS) for further review by human contact tracers. For Bluetooth, the data would go to a Ministry of Health server that would automatically distribute that data to other devices without human intervention.

### Delta Wave (17 Aug 2021 to 6 Jan 2022)

The beginning of the Delta wave was the first real opportunity for digital contact tracing to be used in outbreak conditions in New Zealand. The Delta wave presented a challenge for contact tracing as the number of cases and contacts pushed up against the system capacity, particularly in Auckland where the majority of cases were.

The arrival of the Delta wave significantly increased perceptions of risk amongst the community. QR code scanning increased from the 0.5-1M per day range to the 2-3M per day range, and the number of devices participating in Bluetooth increased from approximately 1.5M to 2.1M.

At the beginning of this period, location information continued to be the primary use of NZCTA data. It was reported that BT was not being used at all, evidenced by a lack of ID keys being uploaded to the central server. The Standard Operating Procedure (267) showed that the protocols left the use of Bluetooth to the case investigator’s discretion.

In September 2021, the government announced that scanning of QR codes would become mandatory. Strictly speaking, the subsequent legislation required a record of a visit to be made, through QR code scanning or otherwise. With risk perceptions continuing to increase, alongside a vaccination push and replacement of the alert level system with the traffic light system, this led to daily QR code scans reaching the 3-4M range during December 2021, and Bluetooth exceeded 2.4M devices. Assuming that most people only have one device and that this was for the population above the age of 16 (the age specified in the privacy impact assessment),^3^ this represented a participation rate of approximately 60%, which was one of the highest rates of any jurisdiction globally with voluntary participation.^4^

Around this time, the same team responsible for NZCTA at the Ministry of Health were also responsible for designing and developing the infrastructure for vaccination passes, which pulled a lot of the key resources and attention away from NZCTA development.

### Omicron Wave (7 Jan 2022 to 15 Feb 2022)

With the arrival of the more infectious Omicron wave in late 2021, the government announced a new strategy after the Christmas/New Year’s break. 7 January 2022 represents the date that the Omicron variant overtook the Delta variant in sequencing results (based on testing of arriving travellers who were generally in quarantine, with community spread only documented in mid-January 2022). This period coincided with a move from PCR testing to RAT testing, which relied on individuals self-reporting their results, along with added error relative to the clinician and laboratory-led PCR process.

In mid-January, MoH developed and tested a self-service contact tracing survey to reduce the burden on contact tracers by allowing those who were able to report their own information. This was separate to NZCTA and delivered as a webform, which asked users to describe their symptoms, identify close contacts where possible, and request additional support if required. Importantly, the survey automatically generated the codes required for users to upload their NZCTA location and BT data, allowing the NZCTA processes to become fully automated.

The length of the survey was criticised, especially considering that individuals were asked to complete it at a time when they were probably most ill. It was intended that there would be follow-up phone calls if a person reported a positive test result but did not complete the form, but as the case numbers increased this support became less practical.

Over the next two months the form was tweaked several times, both to clarify the language used and to re-prioritise the data being collected by changing the order of the questions. For example, at one point the prompt to upload NZCTA data was moved from the beginning of the survey to the end.

### Full Automation (16 Feb 2022 until 2 August - the end of data availability)

From 16 February 2022, initial phone calls by case investigators were replaced for everyone with a texted link to the self-service survey. Around this time, manual contact tracing nationally shifted to focusing only on high-risk locations, such as hospitals and aged care facilities, tracing clusters rather than all individual cases.

From 25 March 2022, the government announced that scanning QR codes was no longer required. This step came after a month of decline in scan rates, as many individuals felt that there was no point in continuing to use the app. NZCTA essentially became Bluetooth only with 2.3M devices still participating.

In March 2022, with QR code/location information used rarely, the BT system was notifying thousands of contacts each day. For every case uploading their BT data, approximately 3-5 contacts were notified of potential exposure. However, due to the privacy design of the BT system, contacts were not informed of who they may have been exposed by, or where they were exposed. An approximate exposure time was given, but due to time zone and configuration issues this could be off by as much as 24 hours, which led to significant confusion for notified contacts.

As we moved through the government’s three-phase Omicron response strategy, isolation requirements for both cases and contacts were gradually reduced, and enforcement waned. These changes limited the impact of digital contact tracing and contact tracing generally, as the intended outcome of these activities is to isolate infected individuals (who have not yet become known cases) from the community to reduce onward spread of the disease.

Table 1. Isolation requirements for cases and contact at different periods in 2022

| **Phase** | **Date From** | **Cases** | **Contacts** |
| --- | --- | --- | --- |
| Phase One | 26 January 2022 | 14 days | 10 days |
| Phase Two | 16 February 2022 | 10 days | 7 days |
| Phase Three | 24 February 2022 | 10 days | 7 days (household only) |
|  | 11 March 2022 | 7 days | 7 days (household only) |
|  | 12 September 2022 | 7 days | 5 days of daily testing for household contacts |

## Appendix 2 Data Dictionary

The datasets that have been released by the Ministry of Health.

### Case demographics

This dataset contains data about the demographics and participation in digital contact tracing systems for every reported case of COVID-19 in New Zealand from the National Contact Tracing Solution (NCTS). To our understanding this includes community cases only and excludes cases at the border or in Managed Isolation and Quarantine (MIQ).

| **Field Name** | **Type** | **Description** | **Codes/Values/Notes** |
| --- | --- | --- | --- |
| case_id | text | An incrementing value for each case starting at 1. | In the format “CS-[NUM]” |
| case_created_datetime | datetime | When the case was created in NCTS, either manually by a contact tracer or automatically based on the case reporting via My COVID Record. | “YYYY-MM-DD HH:MM:SS” |
| case_investigation_datetime | datetime | When case investigators/contact tracers began tracing the case. | “YYYY-MM-DD HH:MM:SS” |
| start_date_of_infectious_period | date | The estimated date for when the case became infectious, derived as two days prior to symptom onset or the date of case creation. | “YYYY-MM-DD” |
| self_service_survey_submitted | boolean | Whether or not the self-service survey was submitted. Survey added on 2022-02-16. | TRUE or FALSE |
| self_service_survey_submitted_datetime | datetime | When the self-service survey was submitted (if available). Field added 2022-03-22. | “YYYY-MM-DD HH:MM:SS” |
| self_service_survey_sent_datetime | datetime | When the self-service survey was sent to the case. Surveys were automatically sent out once a case was self-reported via My COVID Record after 2022-02-16. | “YYYY-MM-DD HH:MM:SS” |
| case_eth | text | Prioritised ethnicity, only one value is given based on National Health Index (NHI). | Māori, Pacific Peoples, European, Asian, MELAA, Residual Categories, Other,  Unknown |
| case_age | text | Age given in five-year brackets at the date of case creation in NCTS (based on NHI). | From ages 0-4 to 120-124 in five-year increments. |
| case_sex | text | Sex of the case (based on NHI). | Male, Female, Unknown, Other. |
| preffered_language | text | Preferred language of the case. |  |
| phu | text | The contact tracing organisation that was responsible for the case. | Standardised fields for 12 Public health units (PHUs) plus three NITC fields. “NITC – CBG” refers to the telehealth provider contracted to provide a national telehealth case investigation service. |
| manual_cc | integer | Number of manually entered close contacts. | Based on the close contact definition at the time. |
| manual_cac | integer | Number of manually entered casual contacts | Based on the casual contact definition at the time. |
| bluetooth_token_generated | boolean | Indicates if a Bluetooth Token (code for uploading Bluetooth keys) was generated/provided to the case by a human contact tracer.  This variable returned false if a case used the self-service survey, even if they did upload their Bluetooth keys. | TRUE or FALSE |
| contact_tracer_app_token_generated | boolean | Was a CTA Token (code for uploading locations) generated/provided to the case. | TRUE or FALSE |

### NZ COVID Tracer Usage

Since the deployment of NZ COVID Tracer on 20 May 2020, the app has been providing analytics on the usage of the app. This is a simple analytics ping when certain events are triggered in the app, with no identifying information passed through to MOH in order to preserve privacy. The data is provided in UTC as the backend technical systems use that as the time zone for consistency. This means that there is some variation with daylight savings, and it is difficult to make a direct comparison to other time-series data as there is a ~12 hour offset. The data is available through to 2022-07-19.

| **Field Name** | **Type** | **Description** | **Codes/Values/Notes** |
| --- | --- | --- | --- |
| Date/Time From | datetime | The start date/time of the reporting period. | “DD/MM/YYYY HH:MM” in UTC |
| Date/Time To | datetime | The end date/time of the reporting period. | “DD/MM/YYYY HH:MM” in UTC |
| App Registrations | integer | The number of times the NZ COVID Tracer app had been downloaded and installed. | Appears to be rounded to the nearest hundred or thousand on some days but not on others. |
| QR Codes Generated | integer | The number of QR codes generated. | Each QR code contained a unique Global Location Number (GLN). |
| NZBN Registered Businesses | integer | The number of businesses with a New Zealand Business Number that generated QR codes. | NZBN registered businesses already had a GLN from the MBIE database, and were a proxy for the number of shops and public venues that needed QR codes – this data appears to be initially unreliable but gains reliability later. |
| Scans | integer | The number of QR code scans during the reporting period. | Each QR code scan is counted once, multiple scans of the same code are counted multiple times. |
| Manual Entries | integer | The number of manual entries during the reporting period. | Manual entries were not initially available in the app as the designers were worried that it would not be tied to a GLN – the feature was later added around July/August 2020 to support users keeping their location diary in one place. |
| Active Devices | integer | The number of unique devices/apps that scanned a QR code or made a manual entry during the reporting period. | Data is available from 2020-06-23, and was a useful metric for understanding how many people were actually participating, and also for calculating a proxy for average mobility. |
| Bluetooth Active (24hr) | integer | The number of devices with Bluetooth Tracing enabled during the reporting period. | Data is available from 2020-12-17, and is determined based on devices checking the central server for Bluetooth keys. There are some issues with smartphone operating systems applying aggressive power saving policies and disabling apps that have not been opened in recently, leading to gradually declining participation without user intervention. |

###

### NZ COVID Outbreak Report

The Ministry of Health produced an automated report to provide an overview of usage of NZ COVID Tracer notification functions on a daily basis. Data is available between 2021-02-25 to 2022-08-02.

| **Field Name** | **Type** | **Description** | **Codes/Values/Notes** |
| --- | --- | --- | --- |
| Date (NZT) | date | The date of the reporting period. | “DD/MM/YYYY” in NZST. |
| Location alerts | integer | The number of location-based notifications generated. | One exposure event could generate multiple notifications, as each device independently checks for matches with their diary locally. |
| Location alerts - devices | integer | The number of unique devices that generated alerts that day. | Assuming that one device corresponds to one person, this deduplicates people who receive multiple alerts in one day (e.g. a family member who was at all of the same places as a case). |
| Callbacks requested | integer | The number of times users asked for a Healthline callback based on a location alert message that day. | This was a very rarely used feature as a flag had to be set on the Location when the alert was sent out in order to enable this function. Most alert messages asked users to call Healthline if they needed advice. |
| Callbacks requested - devices | integer | The number of unique devices that asked for callbacks that day. |  |
| Bluetooth alerts - devices | integer | The number of devices that received Bluetooth Tracing-based exposure notifications that day. | Since the keys rotate regularly, if two people were in close proximity for a long time then it is possible that Bluetooth Tracing would generate multiple matches throughout a day without necessarily knowing that they were connected, hence they needed to be aggregated at the daily level. |
| Bluetooth key uploads - devices | integer | The number of devices that uploaded their Bluetooth Tracing keys that day. | Whereas other fields related to contacts, this field relates to cases who voluntarily uploaded their Bluetooth Tracing keys. This became much more common once codes to release Bluetooth Tracing keys to the central server were provided automatically through the self-service survey. |

### COVID Tracer App locations

This dataset contains locations provided voluntarily by cases, either from scanning QR codes or manual entries. Only contact locations (CL) that were significant were investigated or coded, so the vast majority have no contextual or description information beyond the fact that a location was uploaded. Human contact tracers made decisions about whether or not to designate a contact location as an “exposure event” based on the risk of exposure (low/medium/high risk, with sensitive or completed locations (e.g. households) excluded).

| **Field Name** | **Type** | **Description** | **Codes/Values/Notes** |
| --- | --- | --- | --- |
| case_id | text | In the format “CS-[NUM]” where [NUM] hopefully corresponds to the same case in the case demographics dataset. | |
| contact_location_id | text | In the format “CL-[NUM]” where [NUM] is an incrementing value. This does not appear to correspond to the Global Location Number (GLN) for QR codes. | |
| contact_location_created_datetime | datetime | When the contact location was created in NCTS. In the format “YYYY-MM-DD hh:MM:SS am/pm” | |
| ee_created_from_contact_location | boolean | Was the location designating an “exposure event” based on clinical judgement of contact tracer. | |
| exposure_event_created_datetime | datetime | When the exposure event was created in NCTS in the format “YYYY-MM-DD hh:MM:SS am/pm” | |
| exposure_event_start_datetime | datetime | When the exposure event started in the format “YYYY-MM-DD hh:MM:SS am/pm”, usually derived from interview or QR code scan time. | |
| exposure_event_end_datetime | datetime | When the exposure event ended in the format “YYYY-MM-DD hh:MM:SS am/pm”, usually derived from interview context with a buffer. | |
| exposure_event_management_organisation | text | Contact tracing organisation responsible for managing the case and their locations. Standardised fields for 12 PHUs plus three NITC fields and the National Close Contact Service (NCCS) for overflow. “NITC – CBG” refers to the telehealth provider contracted to provide a national telehealth case investigation service – reported as NCIS on advice from the Ministry of Health. | |
| exposure_event_gathering_type | text | Contact tracer coded categorisation of the venue. Categories included Other, Retail Store, Healthcare Facility, Hospitality, Contact Location [?], Supermarket, Social Gathering, Recreation/Leisure, School or University, Residence, Public Transport, Work, Flight, Other Transport, Private Gathering | |
| exposure_event_gathering_subtype | text | Contact tracer coded categorisation of the venue. Added from 2021-10-22 onwards:  Other, Other Locations, General Retail, Supermarket, Place of Worship, Eatery or Food Outlet, Gym, Restaurant, Aged Residential Care Facility, Bar, School, Hospital, Café, Mall, Corporate Office, General Practice, Petrol Station, Dairy or Superette, Early Childhood Learning Centre, Pharmacy, University, Organised Events, Factory, Hardware Store, Private – Visit to Another Household, Grocery Store, Temporary Accommodation, Police or Prison, Train, CBAC, Taxi or Rideshare, Marae, Bus, Private Vehicle, Laundromat, Cinema, Dental Practice, Park or Playground, Domestic Flight, Student Hall, Beach, Private – Visit to My Household, Apartment Building | |
| exposure_event_for_casual_contacts | boolean | Was the exposure event considered relevant for casual contacts (or only close contacts). Of the 3690 locations, only 32 were considered relevant for casual contacts. | |
| published_as_loi | boolean | Was the exposure event published on the Locations of Interest (LoI) list on the MOH website. Added to NCTS during August 2021 (Delta) outbreak, a total of 861 exposure events were published as LoIs. | |
| public_loi_start_datetime | datetime | Start date/time of LoI published on MOH website. Format “YYYY-MM-DD hh:MM:SS am/pm” Added to NCTS during August 2021 (Delta) outbreak. | |
| public_loi_end_datetime | datetime | End date/time of LoI published on MOH website. Format YYYY-MM-DD hh:MM:SS am/pm.” Added to NCTS during August 2021 (Delta) outbreak. | |
| public_loi_name_of_event | text | Name of the event or location. Generally the name of the venue/location. | |
| public_loi_location | text | Unique location ID. Origin of the ID structure is unclear. | |
| public_loi_website_advice | text | The text of the advice given on the MOH website. Added to NCTS during August 2021 (Delta) outbreak, advice was based on whether the location was a close, casual, or later casual plus contact exposure event. | |
| push_notification_sent | boolean | Was the exposure event published to NZ COVID Tracer apps to check against local diaries and generate notifications. | |
| push_notification_created_date | datetime | Date/time when the notification was generated by PHU/NITC. Format “YYYY-MM-DD hh:MM:SS am/pm.” | |
| push_notification_message | text | The text of the advice given in the push notification. Messages may not match the LoI website advice exactly, generally shorter and with a notice to call Healthline for advice. | |

## Supplementary Table 1: Comparison of location generation by ethnicity, stratified by COVID-19 phase and contact tracing organization

| **Characteristic** | **Overall** | | | **Delta** | | | **Omicron** | | |
| --- | --- | --- | --- | --- | --- | --- | --- | --- | --- |
|  | **cases** | **Tokens** | **%** | **cases** | **Tokens** | **%** | **cases** | **Tokens** | **%** |
| **Māori** |  |  |  |  |  |  |  |  |  |
| PHU | 3688 | 514 | 13.9% | 3110 | 422 | 13.6% | 578 | 92 | 15.9% |
| NCIS | 293 | 120 | 41.0% | 170 | 52 | 30.6% | 123 | 68 | 55.3% |
| **Pacific** |  |  |  |  |  |  |  |  |  |
| PHU | 2492 | 363 | 14.6% | 2184 | 299 | 13.7% | 308 | 64 | 20.8% |
| NCIS | 2185 | 767 | 35.1% | 142 | 44 | 31.0% | 2043 | 723 | 35.4% |
| **Asian** |  |  |  |  |  |  |  |  |  |
| PHU | 551 | 203 | 36.8% | 319 | 111 | 34.8% | 232 | 92 | 39.7% |
| NCIS | 1454 | 831 | 57.2% | 156 | 111 | 71.2% | 1298 | 720 | 55.5% |
| **Other** |  |  |  |  |  |  |  |  |  |
| PHU | 1681 | 487 | 29.0% | 1364 | 344 | 25.2% | 317 | 143 | 45.1% |
| NCIS | 1284 | 652 | 50.8% | 324 | 153 | 47.2% | 960 | 499 | 52.0% |

## Supplementary Table 2. Conversion of exposure events into push notifications by location

| Location | Exposure events | Push notifications | Conversion percentage |
| --- | --- | --- | --- |
| All | 2630 | 840 | 31.9% |
|  |  |  |  |
| Other | 813 | 325 | 40.0% |
| Retail Store | 443 | 134 | 30.2% |
| Supermarket | 329 | 169 | 51.4% |
| Contact Location | 322 | 101 | 31.4% |
| Hospitality | 247 | 37 | 15.0% |
| Healthcare Facility | 141 | 31 | 22.0% |
| Recreation/Leisure | 88 | 27 | 30.7% |
| Social Gathering | 83 | 2 | 2.4% |
| School or University | 72 | 13 | 18.1% |
| Public Transport | 48 | 0 | 0.0% |
| Work | 44 | 1 | 2.3% |

## Supplementary Figure 1: Location token generation among cases by ethnicity (A), age group (B), contact tracing organisation (C) and sex (D)


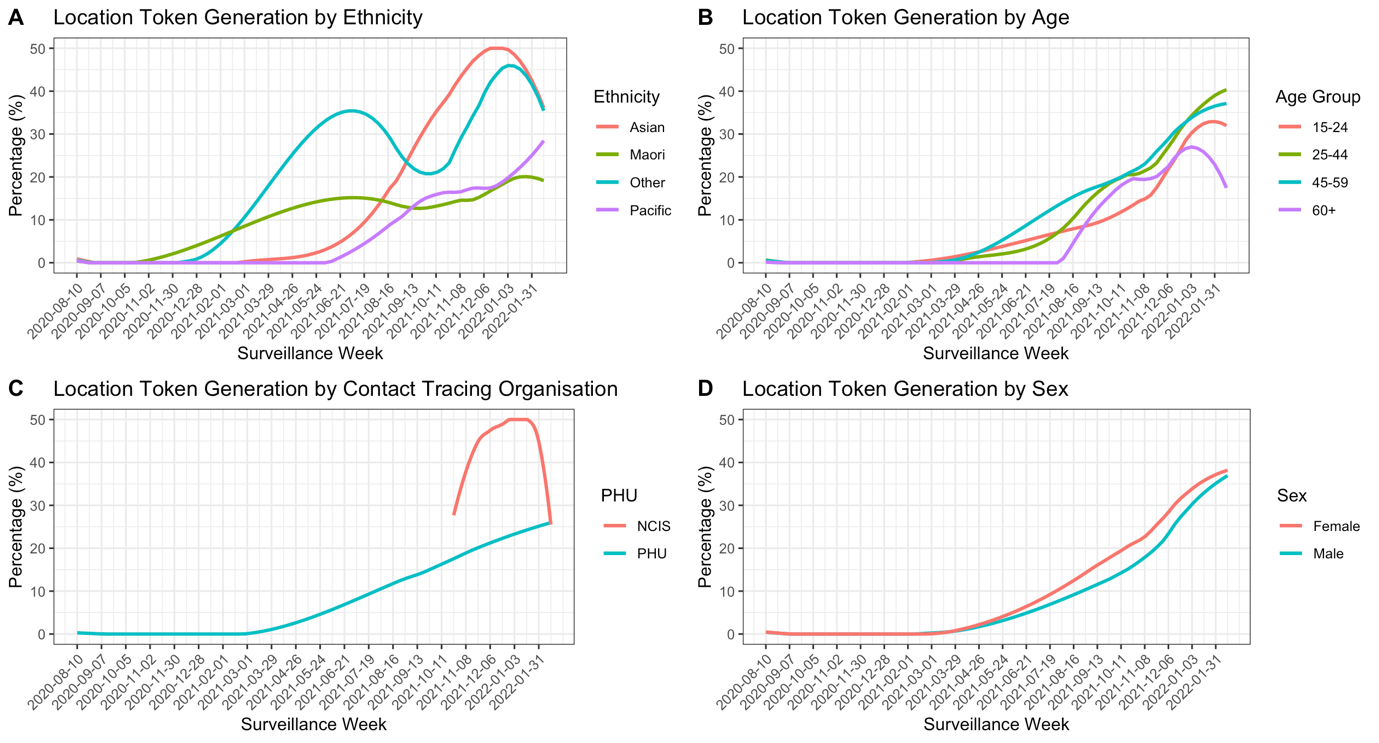

Supplement: Chambers et al. supplementary material [file S0950268824000608sup001.docx]
